# Impacts of Enrichment Type (Hut vs. Platform) on Chronic Stress and Anxiety in Six Fast- and Slow-Growing Broiler Strains

**DOI:** 10.3390/ani16111657

**Published:** 2026-05-29

**Authors:** Alexandra Ulans, Leonie Jacobs

**Affiliations:** 1 Prestage Department of Poultry Science, North Carolina State University, Raleigh, NC 27607, USA; aculans@ncsu.edu; 2 School of Animal Sciences, Virginia Tech, Blacksburg, VA 24061, USA

**Keywords:** anxiety, attention bias, animal welfare, chronic stress, environmental enrichment, feather corticosterone, secretory immunoglobulin-A, slow-growing broiler

## Abstract

The objective of this study is to assess anxiety and chronic stress in fast- and slow-growing broilers when housed with a hut or platform enrichment. We obtained 264 day-old chicks each of Cobb 500, Ross 308, and Ross 708, Redbro-Yield, Redbro M, and Ja57NH strains, and placed them by strain across 72 pens (22 birds/pen) with an A-frame hut or a perforated platform with ramps. Anxiety was assessed via the attention bias test (proportion of birds feeding, vigilance), and chronic stress was assessed through feather corticosterone (fCORT) and secretory immunoglobulin-A (SIgA) concentrations in excreta at 3.7 kg body weight. More Ja57NH broilers began feeding during the test compared to Cobb 500 (*p* = 0.037) and Ross 708 (*p* = 0.033) broilers. Redbro M broilers were less vigilant than Ross 308 broilers (*p* = 0.038). Redbro M broilers had lower fCORT concentrations than Ja57NH broilers (*p* = 0.019). Redbro M and Ja57NH broilers had lower SIgA concentrations than all fast-growing strains. Enrichment type had no impact on anxiety or chronic stress responses. Both Ja57NH and Redbro M broilers showed improved indicators of affective state (less vigilance, tended to feed more during the test, lower fCORT concentrations) compared to other strains assessed at market weight. SIgA levels seemed strongly impacted by genetics, so SIgA may not be appropriate as an inter-strain comparison of chronic stress. The two fastest-growing broiler strains were genetically predisposed to greater anxiety compared to the two slowest-growing strains, which has implications for their welfare.

## Simple Summary

This study explored whether fast- and slow-growing broiler strains and single environmental enrichment types influence anxiety and chronic stress. In this study, six strains of broilers with differing growth rates were reared, housed with either an A-frame hut or a perforated platform, and their anxiety and chronic stress responses were assessed. This study finds that slow-growing Ja57NH and Redbro M broilers are less anxious than fast-growing Cobb and Ross lines, and that Redbro M broilers also show lower long-term stress hormone levels. Overall, genetic strain has a stronger impact on broiler welfare indicators than enrichment type.

## 1. Introduction

Fast-growing broilers are genetically selected for high productivity, with high average daily gain and low feed conversion rates. However, their fast growth can result in poor animal welfare outcomes [1,2,3,4,5,6]. While fast-growing broilers can show worse health outcomes [1,2,3,4] and reduced expression of natural behaviors [5,6], little is known about differences in their affective states. As affective states reflect the long-term emotional experiences of an animal, measuring them provides direct insight into overall welfare [7].

Some experiments have assessed differences between strains, but the information available is still limited. With regard to anxiety, one fast-growing broiler strain (Ross 708) was more anxious than one slow-growing strain (Redbro M) [8], but no other strains have been compared. With regard to fearfulness, one fast-growing strain (Ross 308) was less fearful than three slow-growing strains (Redbro, Rowan Ranger, JA57) [9,10,11]. No differences in chronic stress between a fast-growing (Ross 708) and slow-growing strain (Redbro M) were observed when measured via feather corticosterone levels [12]. These inconsistent findings highlight the need for broader multi-strain comparisons. However, no studies have compared affective states across multiple fast- and slow-growing broiler strains, limiting our knowledge of how genetic strain drives welfare outcomes. Slow-growing broilers have been subject to more diverse selection pressures, often prioritizing traits such as robustness and animal welfare [6]. These differences in genetic selection may influence how broilers perceive and respond to their environment, potentially shaping their affective states. Without a clear understanding of these potential differences, it is difficult to make informed, welfare-oriented decisions in breeding and management practices.

Genetic selection related to growth rate may play an important role in broiler chickens’ emotional states. Animal welfare outcomes have been compared across strains with differing growth rates, but strain identities were anonymized or omitted [5,13,14,15]. With few slow-growing strains studied, it is important to continue this investigation because they are being considered for large-scale commercial production. By examining how genetic strain influences affective states, we can develop strategies to ensure both good productivity and good animal welfare in commercial settings.

Even though slow-growing broilers show improved welfare outcomes compared to fast-growing broilers, slow-growing strains still experience similar welfare concerns, just to a lower degree [5,15,16]. One way to improve animal welfare for both fast-growing and slow-growing strains is to provide access to complex environments through environmental enrichments [17,18,19]. Platform perches can benefit fast-growing broiler chickens’ welfare by reducing the prevalence of leg disease [20,21], footpad dermatitis severity [17], and fear [22]. The same impact was observed for gait abnormalities in slow-growing broilers [21,23]. Platform access can also increase the frequency of natural behaviors, including standing, resting, locomotion, and comfort behaviors in fast-growing broilers [24]. Providing shelters, such as A-frame huts, promotes resting behavior [25] and can function as a barrier that birds prefer to sit by compared to sitting in open space [26,27]. Barriers have similar features to huts (without the dark space underneath) and can reduce the frequency of aggression, disturbances [28], and the severity of footpad dermatitis [29].

The animal welfare benefits of platform perches [16,30,31,32,33,34] and barriers [35,36] were assessed in conjunction with other resources to create complex environments. This makes it difficult to discern what benefits the platform perches or barriers provide individually. Measuring a resource’s potential to improve welfare can allow producers to make practical choices for their flocks. Platform perches and huts have potential for commercial application due to their light weight and low cost, thereby minimizing effort and increasing the likelihood of adoption by producers. However, it is unclear whether these resources have different impacts on broilers’ affective states.

Affective state reflects coordinated activity across cognitive bias, neuroendocrine stress physiology, and immune modulation. To get a holistic view of affective state, both physiological and behavioral indicators can provide insights. Anxiety is a negative affective state with detrimental impacts on animal welfare [37]. Anxiety in chickens is assessed using an attention bias test, which quantifies how long it takes for animals to divert their attention from a negative stimulus (threat) towards a positive stimulus (reward) [38,39]. A prolonged focus on the negative stimulus is indicative of greater levels of anxiety, thus poorer welfare [38,39]. A complex environment lowered anxiety in fast-growing broilers compared to a simple environment [40]. Slow-growing Redbro M broilers were less anxious than fast-growing Ross 708 broilers regardless of housing environment [8]. The impact of singular resources (platform perches and huts) on anxiety across multiple genetic strains has not yet been studied.

Chronic stress can cause a negative affective state and is therefore detrimental to animal welfare [41,42]. Besides direct negative effects, chronic stress results in decreased cognitive ability [43], growth [44], and immune function [45]. Feather (f) corticosterone (CORT) concentration is a validated non-invasive measure of chronic stress in broilers [41,42]. As feathers grow, the vascularized sections incorporate non-functional components, including circulating CORT, within the keratin structure [46,47]. Higher concentrations of CORT in the blood will lead to more CORT being deposited in the feather [41,42,46]. fCORT concentrations can illustrate the total accumulation of acute stress responses experienced over the feather’s growth period [46,48,49] and thus can be a non-invasive tool to quantify physiological stress. fCORT positively correlates with other stress measures, such as heterophil-to-lymphocyte ratios and serum CORT concentrations [42], further confirming its validity. Previously, we assessed fCORT concentrations as a measure of chronic stress in broilers but found no differences between environmental complexity treatments or between a fast-growing (Ross 708) and a slow-growing strain (Redbro M) [12]. However, results may differ when boilers are housed with a singular resource or for other strains, warranting further investigation.

Because chronic stress suppresses immune system activity, measures of immune function have the potential to be used to quantify chronic stress levels too [50]. Secretory immunoglobulin-A (SIgA) is an antibody found on mucosal surfaces and can be used as an indicator of mucosal immune system functioning [51,52]. SIgA concentrations show promise as biomarkers for animal welfare, as they increase with positive experiences and decrease with negative experiences [53,54,55,56,57,58]. This may provide insights into the emotional valence of the animal’s experience, unlike glucocorticoid measures [59]. Low SIgA concentrations can indicate immunosuppression from chronic stress [59]. For instance, chronic heat stress resulted in low plasma IgA levels in fast-growing broilers [60] and laying hens [61]. A low-complexity environment resulted in low plasma IgA concentrations in fast-growing broilers [62], while housing conditions had no impact on SIgA concentrations in Bovans Brown laying hens [63]. The exploration of differences in chronic stress responses through fCORT and SIgA concentrations could provide more insight into the affective experience of broilers with differing growth potential.

Our objective was to determine the differences in anxiety and chronic stress responses between three fast-growing and three slow-growing broiler chicken strains when raised with either a platform perch or a hut. We predicted that all slow-growing broiler strains would have lower anxiety levels and reduced chronic stress responses (low fCORT, high SIgA concentrations) compared to fast-growing broiler strains. However, SIgA responses may vary depending on immune allocation. We also predicted that broilers raised with a platform would have less anxiety and chronic stress compared to those raised with a hut since it allows for the performance of a highly motivated behavior (perching) and likely will improve leg strength. Furthermore, the provision of an elevated space may allow birds to feel more secure.

## 2. Materials and Methods

### 2.1. Ethical Statement

All animal procedures were approved by the Virginia Tech Institutional Animal Care and Use Committee (IACUC) and were conducted in accordance with institutional guidelines for the care and use of animals in research (protocol #23-015). This experiment took place from 6 March through 4 June 2023 and was reported in accordance with the ARRIVE guidelines.

### 2.2. Animals and Housing

**Figure 1:**
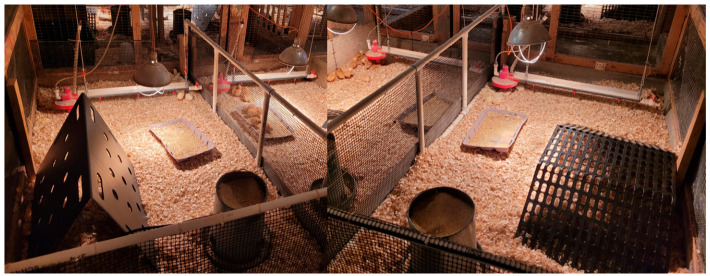
Images of the pen environment for birds raised with either an A-frame hut (**left**) or a platform perch (**right**).

This experiment used a 6 × 2 factorial approach in a randomized block design (house section, chosen through a random number generator), with each strain × enrichment type combination present in each block. Broiler chicken strain and enrichment type were factors at the pen level. We obtained 1584 unsexed Cobb 500, Ross 308, Ross 708, Redbro-Yield, Redbro M, and Ja57NH day-old chicks (264/strain) from a hatchery (South Fork, PA, USA), where they were vaccinated for Marek’s disease and transported to the research facility. Upon arrival, chicks were separated by strain, and 22 chicks were randomly selected to be placed in randomly selected pens across six blocks, totaling 72 pens (Figure 1), resulting in 6 replicates per treatment (strain × enrichment combination). This created a projected stocking density of about 28 kg/m^2^ to replicate commercial settings while still allowing room for the enrichments.

Pens (1.2 × 2.4 m) contained new pine shavings at approximately 6 cm in depth, with one hanging galvanized cone feeder (similar to the Galvanized Cone Feeder, Premier 1, Washington, IA, USA) and an automated water line with three nipple drinkers (Ziggity Systems Inc., Middlebury, IN, USA). Broilers had ad libitum access to feed and water. The corn-soybean meal diets were the same for all strains and were prepared according to the nutritional specifications for conventional fast-growing broilers, split into three feeding phases: starter (CP 23%, ME 3000 kcal/kg), grower (CP 21%, 3100 kcal/kg), and finisher (CP 20%, 3150 kcal/kg). The broilers received these diets for roughly one-third of their production period. This minimized diet as a confounding factor, since “exposure” was balanced over time. Fast-growing broilers received starter feed from day 0 to 18, grower feed from day 18 to 34, and finisher feed from day 34 to processing weight. Slow-growing broilers received starter feed from day 0 to 24, grower feed from day 24 to 57, and finisher feed from day 57 until target market weight. The house temperature was set at 35 °C on day 0, gradually reduced to 21 °C by day 14, and remained at 21 °C until the end of the trial. Temperature was managed automatically and ensured through twice-daily checks at the bird level. We applied an artificial lighting schedule of 24L:0D for the first 3 days using heat lamps, then 18L:6D until the end of the experiment, with a light intensity of approximately 5 lux during light hours to simulate commercial conditions [64].

### 2.3. Genetic Strains

Fast-growing Cobb 500 [65], Aviagen Ross 308 [66], and Aviagen Ross 708 [67] broilers are commonly used in the United States’ large-scale commercial broiler chicken production. They are broiler chicken strains that rapidly gain weight, with an average daily gain of >77 g/day in this experiment (Table 1). The slow-growing strains have a growth rate of <52 g/day in this experiment (Table 1), and none of these strains are used in large-scale production in the United States. Redbro-Yield originates from Hubbard Redbro M females [68] × Hubbard Color Yield males [69]. The slow-growing Redbro M is the offspring of Hubbard Redbro M females [68] × Hubbard Redbro M males [69]. The slow-growing Ja57NH is the offspring of Ja57ki females [68] × New Hampshire males. The New Hampshire strain was not developed by a genetics company but by researchers at the New Hampshire Agricultural Experiment Station and farmers early in the 20th century [70]. This strain was developed from the Rhode Island Red chicken [70].

### 2.4. Resources

**Figure 2:**
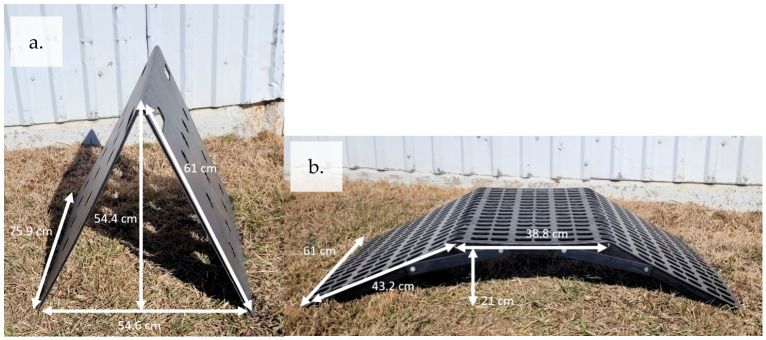
(**a**) Image and corresponding dimensions of the A-frame hut. (**b**) Image and corresponding dimensions of the platform perch.

Broilers were housed with either an A-frame hut (Figure 2a) or a platform perch (Figure 2b). Huts were made of corrugated plastic with holes in a grid-like pattern on the sides. The platforms with ramp access were made of solid plastic and had a slatted design, with a ramp angle of 29°. All strains of broilers at all ages were observed to be able to access the platform.

### 2.5. Measurements

All measurements were conducted when birds reached a target weight of 3.7 kg. All measures and samples were taken on the same day.

#### 2.5.1. Attention Bias Test

The attention bias test was performed at the group level with three birds, similar to that described in [8]. The test was performed with 6 birds per pen (two tests per pen, *n* = 426) when broilers weighed 3.7 kg (Table 1). This number of broilers was chosen as a representative sample for their pen. Tests were performed between 9:00 a.m. and 12:00 p.m. on the testing day for each strain.

Three familiar birds were tested simultaneously in an arena in a separate room of the facility. Testing in groups reduces social isolation and stress, and increases participation within the test [40]. Birds were handled upright and placed in a tote to transport them to the arena for a 30-s walk. The center of the arena had a feeder filled with familiar feed and a liberal amount of mealworms, acting as a positive stimulus. Birds were placed in the arena, and the observer quickly left the room. Once out, the observer introduced the negative stimulus by playing a conspecific alarm call indicating a ground predator for 8 s at approximately 95 dB (FUGOO, Los Angeles, CA, USA) [38].

Latency to begin feeding was recorded live from videos (2.7k, IP Bullet camera FLPB133F, FLIR Systems Inc., Wilsonville, OR, USA) in the room adjacent to the testing arena. A longer latency to feed is used as an indicator of anxiety [38,39]. Using the video recordings, the duration of vigilance behaviors was observed for one of the three randomly chosen birds (*n* = 144) through continuous focal sampling from the beginning of the test until either 300 s had passed or until all three birds began feeding [8,38,40]. Birds were randomly selected by numbering them left to right and using a random number generator to determine which bird to assess. Birds were considered vigilant if they showed at least one of the three vigilance behaviors (Table 2) [71]. Vigilance behaviors were coded by a single trained observer using BORIS software (9.7.15) [72].

#### 2.5.2. Feather Corticosterone Concentration

Three broilers per pen were randomly selected on day 2 of age, wing banded, and marked with livestock marker (All-Weather Paintstik, LA-CO Industries, Inc., Elk Grove Village, IL, USA). When birds reached 3.7 kg of body weight (Table 1), feathers were collected from these broilers (*n* = 18/treatment, *n* = 213 total) to determine feather CORT concentrations as an indicator of chronic stress [41,46]. Primary wing feathers 6 and 7 were collected by cutting the calamus. Broilers were held upright while the wing was stretched out to cut the feathers. Samples were collected between 9:00 a.m. and 11:00 a.m. Feathers were stored in plastic bags (SC Johnson, Racine, WI, USA) at −20 °C until CORT extraction. CORT was extracted from the feathers following a protocol similar to that described in [12,63]. Feathers were weighed (mg), rinsed with water, and then minced into <5 mm-sized pieces [12]. Thereafter, 1 mL of methanol was added, and samples were placed in a sonicating water bath (CO-Z, Shanghai, China) for 30 min (20 °C, 40 kHz). Next, samples were placed in a shaking water bath (Jouan Inc., PrecisionSci. Div., Chicago, IL, USA) overnight to extract CORT (50 °C, 50 rpm). The feather material was separated from the methanol using a vacuum. The methanol was completely evaporated from the filtrate through air drying, 1 mL of ELISA buffer (400060, Cayman Chemical, Ann Arbor, MI, USA) was added, and samples were placed in a −20 °C freezer until assayed. Samples were assayed in triplicate using a commercial enzyme-linked immunosorbent assay kit (501320, Cayman Chemical, Ann Arbor, MI, USA) following the manufacturer’s protocol. If CV% was >15%, the most divergent triplicate was removed [73]. Resulting CORT concentrations were divided by feather weight to express CORT concentrations as ng of CORT/g of feather. Feather weight was chosen over feather length to calculate relative fCORT concentration, as this is considered a more accurate measure [74,75,76]. The final intra-assay CV% was below 15% for all the feather CORT samples (range: 0.5–14.6%).

#### 2.5.3. Secretory Immunoglobulin-A Concentration

Fecal samples were collected from three birds per pen (*n* = 213) when birds reached 3.7 kg live body weight (Table 1). Freshness of the samples was ensured by visually confirming defecation to prevent degradation of SIgA by fecal proteases. All samples were collected between 9:00 a.m. and 1:00 p.m. on the day of testing for each strain. Following collection, the samples were placed on dry ice and stored in a −80 °C freezer. SIgA was quantified using similar methods to those described in [62]. A total of 1 mL of a saline extraction buffer (0.01 M phosphate-buffered saline, 0.5% Tween (Sigma-Aldrich, St. Louis, MO, USA), and 0.05% sodium azide) was added to each 100 ± 1 mg of fecal sample, followed by manual homogenization. Fecal suspensions were centrifuged at 1500× *g* for 20 min at 5 °C (Centrifuge 5417R, Eppendorf, Hamburg, Germany), and the supernatant was removed and placed in microcentrifuge tubes. Then, 20 µL of protease inhibitor cocktail (P8340, Sigma-Aldrich, St. Louis, MO, USA) was added to the supernatant and homogenized before storage at −20 °C until analysis. The samples were analyzed for SIgA concentrations via a commercial ELISA kit (ab157691, Abcam, Cambridge, MA, USA) following the manufacturer’s instructions. The intra-assay CV% was below 1.1% for all the samples (min: 0.005%; max: 1.1%). Assay results were divided by their sample weight to express SIgA concentrations as ng of SIgA/g of fecal sample. We wanted to ensure that fecal sample water content would not impact the SIgA concentrations. To calculate water content, 100 ± 1 mg of the sample was weighed in an aluminum weigh dish and dried in a forced-air oven (Freas 645, Thermo Electron Corporation, Marietta, OH, USA) at 55 °C for 72 h [77]. Dried samples were weighed, and the difference was used to calculate the water percentage in the sample. We obtained a water-corrected SIgA concentration by multiplying the SIgA concentrations by the proportion of water in each sample.

### 2.6. Statistics

All statistical analyses were performed in R (4.4.2) using the lme4 package (1.1.35.5) [78,79]. Treatments were applied at the pen level; therefore, the pen was considered the experimental unit. Response variables were measured from individual broilers, which were considered the observational units. Linear mixed models (LMMs) and generalized linear mixed models (GLMMs) were used to assess the effects of strain and environmental enrichment as fixed effects. If data could not be transformed to fit a GLMM distribution, then the data were transformed, and an LMM was used (Table 3). The DHARMa package (0.4.7) was used to assess model fit [80]. Pen was included as a random effect in all models, but block was not because of the lack of impact on outcomes or model fit. For the attention bias test model, pen was included as a random factor nested within test round to account for clustering of observations within test sessions across rounds. Interactions between strain and environmental complexity were removed from the models because they were not statistically significant. Statistical significance was set at *p* < 0.05 and trends at *p* < 0.1. Post hoc analysis was done using Tukey HSD testing to control for the family-wise error rate. The data are presented as LSmeans ± SEM unless otherwise noted.

## 3. Results

### 3.1. Attention Bias

#### 3.1.1. Feeding Behavior

**Figure 3:**
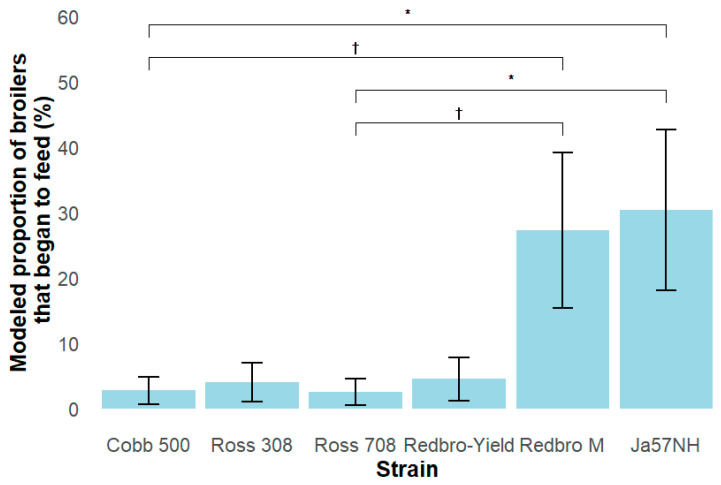
Modeled proportions of broilers (%) that began feeding during the attention bias test by broiler chicken strain at processing age. Error bars represent SEM. Brackets with a * indicate a difference at *p* < 0.05, and a † indicates a trend at *p* < 0.10. *n* = 6 birds/pen, total *n* = 426.

Strains differed in feeding behavior (χ^2^(df = 5) = 16.854, *p* = 0.005, Figure 3). More Ja57NH broilers fed during the test than Cobb 500 (*p* = 0.037) and Ross 708 broilers (*p* = 0.033, Figure 3). There was a trend for more Redbro M broilers to feed during the test than Cobb 500 (*p* = 0.062) and Ross 708 broilers (*p* = 0.054, Figure 3). Feeding behavior did not differ between birds housed with huts or platforms (χ^2^(df = 1) = 0.0002, *p* = 0.989; hut: 7.3 ± 3.4%, platform: 7.3 ± 3.3%).

#### 3.1.2. Vigilance Behavior

**Figure 4:**
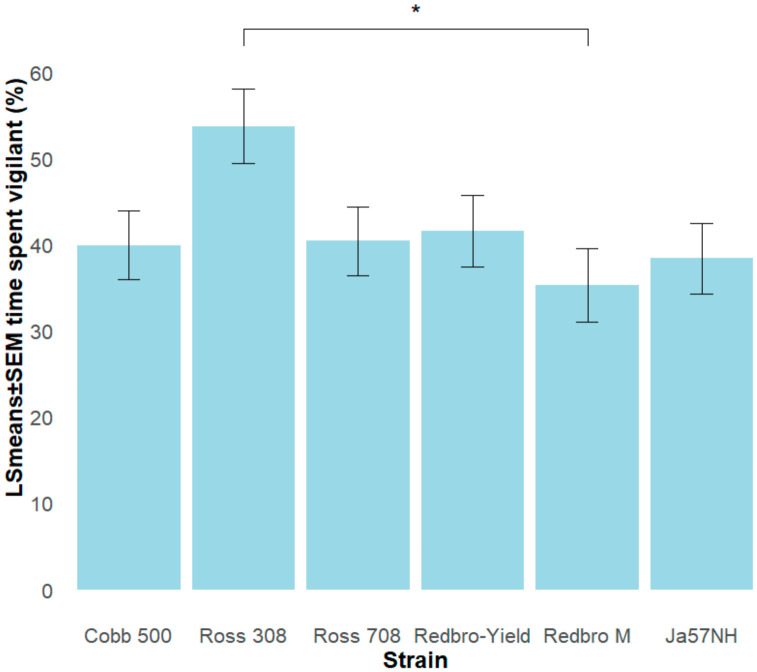
Least squares mean estimates of the proportion of time (%) spent vigilant during the attention bias test by broiler chicken strain at processing age. Error bars represent SEM. Brackets with a * indicate a difference at *p* < 0.05. *n* = 2 birds/pen, total *n* = 142.

Strains showed a tendency to differ in the proportion of time they were vigilant (χ^2^(df = 5) = 10.952, *p* = 0.052, Figure 4), with Ross 308 broilers being vigilant for a greater proportion of time (54%) than Redbro M broilers (35%, *p* = 0.038, Figure 4). Enrichment type did not impact vigilance behavior (χ^2^(df = 1) = 0.122, *p* = 0.727; hut: 42.2 ± 2.4%, platform: 41.0 ± 2.4%).

### 3.2. Feather Corticosterone Concentration

**Figure 5:**
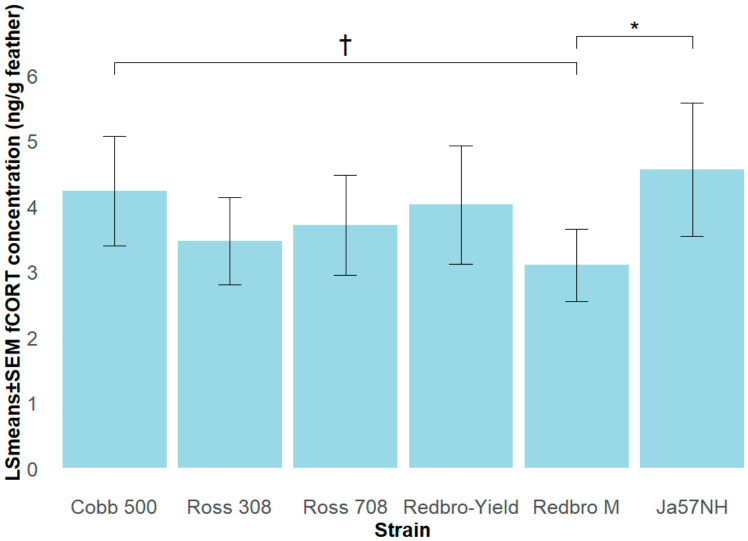
Least squares mean estimates (±SEM) of feather corticosterone concentrations by broiler genetic strain at processing age. Error bars represent SEM. Brackets with a * indicate a difference at *p* < 0.05 and a † indicates a difference at *p* < 0.10. *n* = 3 birds/pen, total *n* = 213.

fCORT concentration varied by strain (χ^2^(df = 5) = 14.865, *p* = 0.011, Figure 5). Redbro M broilers had lower concentrations than Ja57NH broilers (*p* = 0.019). There was a tendency for Redbro M broilers to have lower concentrations than Cobb 500 broilers (*p* = 0.092, Figure 5). Enrichment type did not impact fCORT concentrations (χ^2^(df = 1) = 0.755, *p* = 0.385; hut: 3.77 ± 0.46 ng/g feather, platform: 3.95 ± 0.46 ng/g feather).

### 3.3. Secretory Immunoglobulin-A Concentration

**Figure 6:**
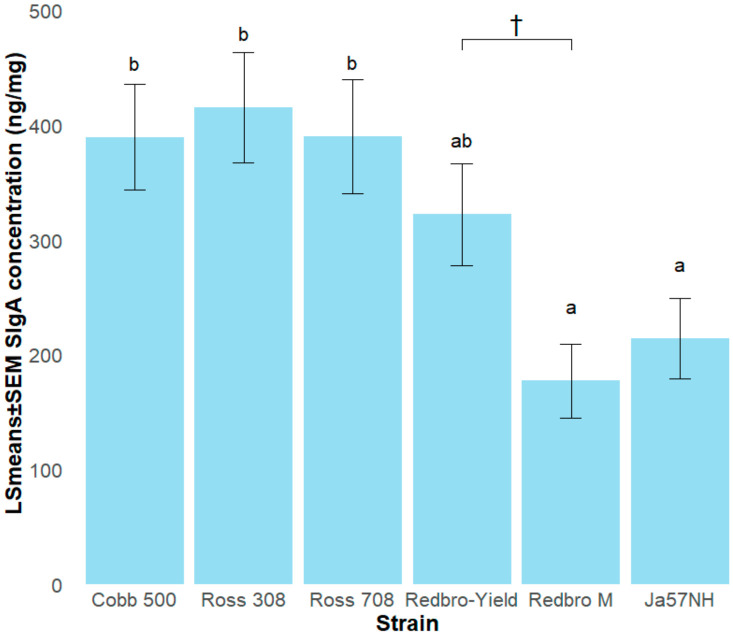
Least squares mean estimates of secretory immunoglobulin-A (SIgA) concentration by genetic strain at processing age. Error bars represent SEM. Bars lacking a common superscript differ at *p* < 0.05. Brackets with a † indicate a difference at *p* < 0.10. *n* = 3 birds/pen, total *n* = 213.

Sample water content averaged 82.2 ± 0.2%. Raw and water-adjusted IgA concentrations were almost perfectly correlated (*r* = 0.999, *p* < 0.001), indicating that adjustment for sample water content had minimal impact on IgA measurements. Due to this, we assessed the raw values moving forward. SIgA concentrations varied by strain (χ^2^(df = 5) = 30.900, *p* < 0.001, Figure 6). All fast-growing strains (Cobb 500, Ross 308, Ross 708) had higher SIgA concentrations than Redbro M and Ja57NH broilers. Redbro-Yield broilers had a trend toward higher SIgA concentrations compared to Redbro M broilers (*p* = 0.081, Figure 6). Enrichment type did not impact SIgA concentrations (χ^2^(df = 1) = 0.244, *p* = 0.622; hut: 319.4 ± 25.2 ng/mg, platform: 302.2 ± 24.0 ng/mg).

## 4. Discussion

This study investigated the differences in affective state in three fast-growing and three slow-growing broiler strains when housed with either a hut or a platform. Whether birds began feeding and the time spent vigilant were indicators of anxiety. fCORT and SIgA concentrations were used as indicators of chronic stress. Some genetic strains may be more prone to these negative affective experiences than others. We observed that the two slowest-growing strains (Ja57NH and Redbro M) were less anxious than one or more fast-growing strains, although vigilance was mostly the same across all strains (except for Ross 308 being more vigilant than Redbro M birds). Chronic stress responses were more variable when considering genetic strain impacts, although there was a clear difference between slow- and fast-growing strains in SIgA levels (low in slow strains). However, as each strain grew at different rates, we cannot untangle what differences were caused specifically by age or growth rate. Environmental enrichment type did not alter anxiety or chronic stress outcomes, suggesting that either the enrichments offered similar benefits or that genetic predisposition dominated the anxiety and stress response under the conditions tested.

### 4.1. Attention Bias

Anxiety is a negative affective state that contributes to the poor overall welfare of animals [81]. Anxiety can be a result of the interactions between genetic susceptibility and environmental factors, although our environmental manipulation (hut or platform) did not impact anxiety. The proportion of broilers feeding shows that they shifted their focus more quickly from the negative stimulus to the positive stimulus, indicating less anxiety [38,39]. The two slowest-growing strains were either more likely to feed or tended to be more likely to feed than the two fastest-growing strains. Although some of these differences were only statistical tendencies, the differences do seem to indicate lower levels of anxiety associated with the genetic predisposition for slow growth. This suggests that genetic factors influence the development of anxiety. This can have a direct effect through heritable traits and an indirect effect by modulating health. Our findings are in line with previous work that demonstrated associations between gene expression and anxiety phenotypes. Ten candidate genes were linked to anxiety-related behaviors in open field tests in domestic chickens and Red Junglefowl [82], and hens carrying a deletion allele were less fearful compared to those with the wild-type allele [83].

Our results indicate that the fastest-growing broilers are likely genetically predisposed to greater anxiety compared to broilers of the slowest-growing strains. The mechanism for this increased anxiety might be indirectly modulated by pain and ease of movement. Fast-growing broilers often experience worse gait compared to slow-growing broilers [6,9,84], and these gait issues are painful, likely causing chronic pain [85,86,87,88]. Chronic pain has been associated with increased anxiety in humans [89,90,91] and rats [92,93,94]. While a connection between anxiety and pain has not been directly assessed in poultry, pain was associated with behavioral and physiological changes indicative of a negative effect [95]. If fast-growing broilers experienced chronic pain, this may explain why they were more anxious compared to Redbro M and Ja57NH broilers. We ensured that the tested birds were able to walk so that they could reach the feed during the test. However, many fast-growing broilers showed at least some deviation from a normal gait while still maintaining walking ability. Even minor deviations in gait have been associated with pain [96]. Since chickens are prey animals, they may attempt to conceal pain as a survival strategy, making pain assessment difficult [97]. It is likely that, even though the fast-growing broilers could walk, they were still experiencing some degree of pain.

Another difference between fast- and slow-growing broilers caused by genetic selection is their body conformation. Fast-growing broilers are selected for high breast meat yield, resulting in a disproportionate increase in pectoral muscle mass located at the front of the body, changing their body conformation and center of gravity [5,98]. This altered morphology is associated with biomechanical changes in gait. Fast-growing broilers walk slower and take wider steps, likely due to the physical demands of the enlarged breast muscle [99,100]. These changes in gait require the broilers to use more energy to locomote [101] and, combined with a high prevalence of gait impairments [102], may reduce their mobility.

Fast-growing broilers, with their altered body conformation and impaired gait, might even experience reduced self-efficacy when navigating potential threats. Self-efficacy is an individual’s confidence in their ability to complete a specific task [103], and a lack of self-efficacy has been associated with anxiety in humans [104,105,106,107,108,109]. If fast-growing broilers perceive themselves as physically unable to escape predators due to compromised mobility, this perceived lack of control could contribute to increased anxiety. Whether this concept applies to poultry is unknown. As self-efficacy was not directly measured in this study, this interpretation remains theoretical and has not been empirically demonstrated.

Overall, these findings are broadly consistent with previous work when accounting for the higher body weight of birds in the present experiment. A previous experiment showed similar findings for anxiety for Redbro M broilers, but not Ross 708 broilers. Redbro M boilers fed at similar rates during the test (39% vs. 36%) [8]. In contrast, Ross 708 broilers fed at a lower rate in the present experiment (10%) compared to the previous experiment (26%) [8]. Broilers in this experiment were heavier (3.7 kg vs. 3 kg), which could have contributed to this difference, since anxiety increased with weight gain [8].

Ross 308 broilers spent more time vigilant than Redbro M broilers, and other strains were equally vigilant during the test, regardless of their access to a hut or platform. This suggests that this behavioral response in the attention bias test was not consistently impacted by genetics related to growth rate or the provided resource. Our prior work indicated that Ross 708 broilers were more vigilant when raised in barren environments compared to Redbro M broilers, but not in complex environments [8]. This suggests that the hut or platform individually did not provide the same benefits as complex environments, as tested in [8]. A single enrichment was either insufficient to meaningfully influence affective states or equally impacted affective states. A complex environment composed of multiple enrichments may be necessary to reduce anxiety in fast-growing broilers. This highlights the importance of considering the function of environmental enrichments when aiming to improve broiler chicken welfare.

Overall, Ja57NH broilers were less anxious than Cobb 500 and Ross 708 based on their likelihood to feed. Redbro M broilers tended to be less anxious compared to Cobb 500 and Ross 708 and were less vigilant than Ross 308 broilers. The three fast-growing strains and the Redbro-Yield broilers showed similar levels of anxiety. Enrichment type did not impact anxiety for any strain. We observed a genetic predisposition for anxiety, with generally higher levels of anxiety, thus a more negative affective state, in the fastest-growing broiler strains.

### 4.2. Feather Corticosterone Concentrations

fCORT concentration measures the amount of stress an animal has experienced over the duration of the feather’s growth [46]. Higher fCORT concentrations indicate that the bird experienced more chronic stress, which negatively impacts its affective state, than those with lower fCORT concentrations [42,46]. While fCORT concentrations did not differ between enrichment types, differences were found between strains. Redbro M broilers had lower fCORT concentrations than Ja57NH broilers and tended to have lower fCORT concentrations than Cobb 500 broilers, indicating less chronic stress experienced by these Redbro M broilers.

Genetic selection for production traits may have favored individuals with lower stress reactivity or better adaptability to commercial environments. For example, Red Junglefowl showed a stronger CORT response to restraint compared to White Leghorn laying hens [110], suggesting that domestication and selective breeding can reduce physiological stress reactivity. However, the differences in fCORT responses among the strains in our study cannot be explained by genetic selection alone. Although the Redbro M broilers have experienced a more relaxed selection for production traits than fast-growing broilers [111], the fast-growing broiler strains did not show lower stress reactivity. In contrast, the dual-purpose Ja57NH strain has not undergone genetic selection pressures for high production, which may explain its higher reactivity [112].

An earlier experiment found no differences in fCORT concentrations between Ross 708 and Redbro M broilers [12]. Still, it was unexpected to see so little difference in fCORT concentrations between the fast- and slow-growing strains, given their differing growth rates, physical conformation, and health outcomes. fCORT may not be a sufficiently sensitive biomarker for detecting chronic, low-level stressors such as persistent discomfort, frustration, or mild pain. While fCORT can reflect long-term HPA (hypothalamus–pituitary–adrenal) axis activation, it may be less responsive to subtle or intermittent stressors that do not elicit strong arousal states [113]. Pain and frustration are not always accompanied by high arousal or elevated corticosterone levels, particularly if the animal adopts passive coping strategies [114,115]. The lack of differences in fCORT concentrations may not indicate the absence of stress or poor welfare, but could reflect limitations in this measure’s sensitivity and specificity under certain conditions. fCORT concentration may need to be used in tandem with other welfare measures to provide a more holistic picture of animal welfare.

### 4.3. Secretory Immunoglobulin-A Concentrations

SIgA concentration reflects mucosal immune activity [50,59]. Chronic stress causes immunosuppression and can result in lower SIgA concentrations [59]. However, SIgA concentrations are influenced by other factors, including growth physiology [116], gut microbiota composition [51], intestinal morphology and absorptive function [52], intestinal integrity or inflammation [117]. Lower levels of SIgA may not necessarily imply immune suppression caused by stress. Redbro M and Ja57NH broilers had lower SIgA concentrations than Cobb 500, Ross 308, and Ross 708 broilers, theoretically indicating immune suppression. This was unexpected, since Redbro M and Ja57NH broilers were less anxious than fast-growing broilers, and Redbro M tended to have lower fCORT concentrations than Cobb 500 broilers. This discrepancy between SIgA levels and behavioral and physiological indicators of stress suggests that strain differences in SIgA levels may not primarily reflect stress-induced immune suppression. Rather, genetic selection for a robust innate immune system in fast-growing strains likely contributed to their high productivity in intensive environments. Thus, genetic differences in mucosal immune investment (physiological resources allocated to the development and maintenance of immune function) between strains could explain the pattern.

Commercial environments expose fast-growing broilers to stressors such as high stocking densities [118,119], variable environmental conditions [120,121,122], and enteric diseases [123,124], which can reduce productivity. Consequently, individuals able to maintain rapid growth despite these challenges may have more robust immune function, potentially resulting in inadvertent selection for greater mucosal immunity and higher SIgA concentrations. Broiler breeding programs are prioritizing disease resistance and vaccine responsiveness [125], which may further contribute to enhanced immune function in these strains. Thus, fast-growing broilers may have been selectively bred for high productivity under commercial conditions that include disease and environmental challenges, which may indirectly favor stronger mucosal immune responses. This could be reflected in elevated baseline SIgA concentrations that are less susceptible to suppression under stressors. If SIgA concentrations are strongly influenced by genetic selection, this biomarker may reflect underlying genetic differences rather than environmental or experiential factors alone. As a result, using SIgA concentrations to compare stress or welfare across genetically distinct broiler strains could be confounded by inherent differences in baseline mucosal immune function.

Enrichment access did not impact SIgA concentrations, similar to the findings reported in [62]. They reported a lower mean SIgA concentration (~76 ng/mg) compared to our findings (~322 ng/mg). This may be caused by differences in the level of complexity (low and high versus one resource in the current study), stocking density (21 kg/m^2^ and 42 kg/m^2^ compared to 27 kg/m^2^ in the current study), and group sizes (90 and 180 birds per pen versus 22 in the current study). Future studies should determine a typical baseline range of SIgA concentrations in broilers.

The higher SIgA levels in fast growers likely reflect an allocation of resources to intestinal immune response rather than a reflection of better welfare. If SIgA concentrations are strongly influenced by genetic selection, particularly in strains bred for enhanced immune function or disease resistance, then this biomarker may reflect underlying genetic differences rather than environmental or experiential factors alone. Therefore, SIgA concentrations may be more appropriate for assessing stress or welfare within a single genetic strain over time or across different environmental conditions, rather than for direct comparisons between genetically distinct strains.

### 4.4. Overall Strain Result Comparison

Affective state responses were consistent across measures for Redbro M but not for Ja57NH broilers. Redbro M broilers tended to be less anxious than Cobb 500 and Ross 708 broilers (proportion of birds feeding), and were less vigilant than Ross 308 broilers. Redbro M broilers also tended to have lower fCORT concentrations, suggesting a more positive affective state across measures. This pattern may reflect fewer distressing experiences throughout production, thus less frequent HPA axis activation [126]. Since these were mostly statistical tendencies, the welfare state of Redbro M broilers compared to fast-growing broilers should be explored further.

Ja57NH broilers were less anxious than Cobb 500 and Ross 308 broilers (proportion of birds feeding), yet their chronic stress response (fCORT levels) was comparable to that of all fast-growing strains. Because anxiety and chronic stress reflect different affective dimensions, it is not unexpected to observe this apparent inconsistency. These outcomes may reflect that Ja57NH broilers are reactive to stressors but can recover effectively [127]. Repeated activation of the HPA axis from short-term stressors may not have translated into anxiety. This implies that Ja57NH broilers have a more positive affective state than fast-growing strains, with Redbro M broilers showing a more positive affective state than both Ja57NH and fast-growing strains.

### 4.5. Enrichment Type

Anxiety or chronic stress outcomes did not differ between the enrichments provided in this study. Both huts and platforms may fulfill a similar behavioral need by offering safety for rest. Huts offer a dark and partially enclosed space that could facilitate resting and comfort behaviors [25]. Platforms offer an elevated perching space that broilers are motivated to access [128]. Our results indicate that both resources had a similar effect on affective states. Previous studies reported mixed outcomes for elevated platforms. Some found no benefit from platform access [29,32,129], while others reported less contact dermatitis [130,131], increased mobility [132], and improved gait [20,133] compared to a control. This may be due to platform design aspects that encourage use or better accommodate the broilers’ physical abilities. In the present study, providing either huts or platforms resulted in similar anxiety and chronic stress outcomes across strains. It is possible that genetic predisposition dominated the anxiety and stress response under the conditions tested.

## 5. Conclusions

Overall, the data suggest that genetic strain is strongly related to anxiety responses, with the two fastest-growing strains being the most anxious compared to the two slowest-growing strains. Enrichment type did not impact this. Redbro M broilers showed reduced chronic stress responses (fCORT) compared to Ja57NH broilers and somewhat reduced (statistical tendency) stress responses compared to Cobb 500 broilers. This confirms improved aspects of affective state in the Redbro M compared to some other strains. The marked differences in mucosal immune function (lower SIgA) between slow-growing strains and fast-growing strains suggest that this biomarker may be influenced by underlying genetic differences. Consequently, SIgA concentrations may be more appropriate for assessing welfare within a single strain across environments or time, rather than for direct comparisons between genetically distinct strains. Animal welfare-centric production systems should prioritize strain selection and biologically meaningful enrichment to achieve measurable improvements in broiler affective states.

## Figures and Tables

**Table 1:** Broiler chicken genetic strains, with age to target weight, mean, and standard error of processing weight and average daily gain.

Strain	Days to Reach 3.7 kg Body Weight	Mean Weight at Testing Point (kg)	Average Daily Gain (g/Day)
Cobb 500	44	3.71 ± 0.05	84.49 ± 1.24
Ross 308	47	3.77 ± 0.04	80.23 ± 0.76
Ross 708	49	3.80 ± 0.04	77.47 ± 1.07
Redbro-Yield	72	3.70 ± 0.03	51.08 ± 0.42
Redbro M	75	3.63 ± 0.03	48.42 ± 0.39
Ja57NH	90	3.45 ± 0.03	38.36 ± 0.36

**Table 2:** Ethogram used to score vigilance behaviors within the attention bias test [8]. All behavioral descriptions were adapted from [71].

Behavior	Description
Erect posture	The bird stands upright, holding its head high (neck not necessarily extended). The head must be above all the neck vertebrae, and the chest must be lifted.
Neck stretch	The neck is elongated either vertically, horizontally, or diagonally, and stretched to its full length. You can typically see skin underneath the feathers when this occurs.
Look	The bird swivels its head to scan the arena. Must turn its head 90° to one side and/or 45° to both sides successively. Look ends when the bird returns its head to a neutral position (front) or initiates any other behavior.

**Table 3:** Summary of data analyses used in this experiment for feeding behavior, vigilance behavior, feather corticosterone (fCORT) concentration, and secretory immunoglobulin-A concentration (SIgA).

Measure	Transformation	Model	Distribution
Feeding behavior	None	GLMM	Binary
Vigilance behavior	None	LMM	Gaussian
fCORT concentration	Boxcox (λ = 0.263)	LMM	Gaussian
SIgA concentration	Square-root	LMM	Gaussian

## Data Availability

Data underlying this manuscript are made accessible through the Virginia Tech Data Repository at https://doi.org/10.7294/32436852.

## Abbreviations

The following abbreviations are used in this manuscript:

fCORT	Feather corticosterone
HPA	Hypothalamus–Pituitary–Adrenal
SIgA	Secretory immunoglobulin-A
